# Medical Teaching and Assessment in the Era of COVID-19

**DOI:** 10.1177/2382120520965255

**Published:** 2020-10-16

**Authors:** Anthony Mark Monaghan

**Affiliations:** Imperial College School of Medicine, Imperial College London, London, UK

**Keywords:** COVID-19, medical education, remote learning, flipped learning, telemedicine, open-book examinations

## Abstract

The coronavirus pandemic has had a significant influence on medical education, most notably in terms of content delivery and provision of assessments. These unique times have facilitated the introduction of many new educational methods in medical schools globally each of which with potential merits and drawbacks. Importantly, the use of remote platforms to carry out online teaching has been especially vital in ensuring the continued training of doctors but other techniques such as telemedicine, online clinical case repositories and even virtual reality headsets are also being used to overcome these difficult circumstances. Some institutions also opted for open book written examinations raising issues surrounding this format’s legitimacy and potential benefits. Practical examinations are even harder to facilitate and although most were cancelled, this crisis may result in innovation which changes their future format. For example some may include audible clinical signs played online through a computer speaker to replace clinical examination. Overall, the COVID-19 pandemic has caused an overhaul of medical school orthodoxy that whilst disruptive, may serve to expose institutions to novel means of teaching and assessment which may ultimately improve medical education in future.

The recent coronavirus pandemic has undeniably had a significant impact on health services globally. However, a further challenge arising from this crisis is the provision of medical education in these unprecedented times. Medical students have had their hospital placements postponed, causing significant anxiety and ushering in a new educational environment which has been difficult to navigate. The major obstacles have included delivering online teaching content as well as adapting means of assessment in such unforeseen circumstances. As a final year medical student at Imperial College London, I am well-placed to comment on the efficacy of these processes during the COVID-19 pandemic. Most pertinently, the insights gleaned will likely have implications for the training of doctors in the future, and as such are of particular interest to the medical education community.

In light of the COVID-19 outbreak, universities were forced to implement several changes to medical education. The primary aim of such changes was to meet students’ learning demands without requiring attendance in-person and platforms such as ‘Zoom’ and ‘Microsoft Teams’ were promptly utilised to allow teaching to be delivered online. These were generally very efficacious in facilitating teaching of essential course content and received positive feedback from medical students at Imperial College. In fact, these remote tutorials often had better attendance than many of their ‘in-person’ counterparts, perhaps owing to the ease and convenience of being able to access them from home. An additional reason for this may be the ease of tracking attendance compared to taking registration in large in-person sessions,^1^ since logging into an online lesson seamlessly creates a digital record of attendance which the facilitator can track. In this way students feel more closely monitored in their participation and are thus more likely to engage. The good student engagement with online learning could indicate that even during conventional times, such methods could be employed alongside traditional teaching to enhance the learning experience. There is evidence that students have a positive perception of online learning methods in both medical and non-medical contexts and that it can be at least as effective as traditional teaching,^2^ further supporting this notion. However, one study during this crisis highlighted some common downsides to remote teaching from the perspective of undergraduate medical students including technical difficulties, easy distraction and some staff being poorly-versed with the technologies used.^3^ Nonetheless, the vast majority of students found online sessions to be a good use of time and the paper proposes at least some incorporation of these into medical teaching, perhaps as part of a ‘flipped-learning’ approach.^3^ Other evidence also suggests that students see online learning as complementing rather than replacing traditional methods, reinforcing the potential for an integrated role in future, perhaps as part of a combined ‘blended-learning’ methodology.^2^ It is possible that current circumstances may simply provide the impetus necessary to drive these teaching techniques into mainstream use.

The provision of medical education is uniquely challenging in that there is a need for vocational exposure in a clinical setting which can’t be sufficiently replaced remotely. Consequently, clinical placements at many medical schools have had to be postponed until the next academic year. However, current circumstances have brought this issue of adaptability to the forefront and much has been done to address it. For example, at Imperial, students were given access to an online bank of patient interviews and interactive cases to supplement clinical study. Furthermore, the use of specially adapted headsets worn by clinicians to give students a ‘first-person’ view of patient examinations was piloted and is intended to be introduced to allow large-scale bedside teaching without overcrowding clinical areas. Another potential solution includes the use of ‘telemedicine’ to allow direct patient interaction via video-conferencing or telephone interviews as has been used at the Mayo Clinic to facilitate teaching during this period.^4^ Such adaptations may prove vital if clinical placements are disrupted again, for example in the event of a coronavirus ‘second wave’. However, it does seem far-fetched, at least in the context of current technology, to suggest they could ever fully replace hospital rotations. Nonetheless, there is evidence that incorporation of ‘telemedicine’ into undergraduate clinical placements may enrich learning by improving core competencies and that it is also well-liked by students.^5^ Another interesting benefit that may emerge in light of this rapid change in educational formats is the nurturing of students’ persistence, resilience and abilities to adapt to sudden changes in circumstances.^6^ These are vital skills to develop as a trainee doctor due to the unpredictable nature of clinical practice and the speed with which seemingly well patients can deteriorate. A flexible mind-set and an ability to calmly and decisively change course when one’s outlook is shifted is invaluable in hospital and may be analogous to having to overhaul one’s learning and revision plan in the face of such seismic educational changes.

In addition to altering teaching provisions, medical schools have also had to adapt their means of assessment. Imperial College London was a world-leader in carrying out completely remote online medical examinations^7^ which students completed under timed conditions at home. These written exams were carried out using an ‘open-book examination’ (OBE) approach. Importantly, there is compelling evidence demonstrating that OBEs significantly decrease student anxiety,^8,9^ a finding reflected in my own experiences during this remote exam season. This is a positive characteristic at any time, but one which is especially important during a global pandemic. Despite this, there were initially concerns regarding the validity of OBEs given their perceived reduced difficulty. However, clinical written examinations are well-suited to OBE formats since questions require nuanced synthesis of lots of information from the clinical scenarios provided and thus the answer cannot simply be searched on the internet. An additional safeguard against cheating is that question order was randomised for each student, rendering collusion between students ineffective. Furthermore, these exams are often very time-pressured (approximately 60 seconds per clinical scenario at Imperial) and there is simply not enough time to intensively research relevant information. Therefore, whilst referring to ‘open-book’ resources does not adequately replace learning and applying important principles, it can instead be used for supplementary facts or to provide a broader context to a scenario. Medicine is more than simply the regurgitation of basic facts and too often examinations at medical school are structured in a way which encourages and rewards this style of learning. OBEs not only prevent students temporarily cramming superfluous information to parrot during assessments but, crucially, also more closely mirror actual clinical practice where such information is easily acquired from resources in hospital. In this way, OBEs allow students to study in a way which is more aligned with life on the wards. An example of some knowledge which could be looked-up during OBEs may include the normal range for reticulocyte count in a child of a particular age or certain clinical status. Without having to spend time committing this dull and dense information to memory, students are able to focus their learning more judiciously on broader and more fruitful principles which are essential to effective practice in hospital. For example, they can instead more intensively revise diagnostic approaches to particular clinical presentations in the knowledge that more esoteric information, such as exact cut-offs for quantitative investigations, can be looked-up as required. This is often exactly how doctors operate in practice and assessment at medical school should seek to mirror this.

Clearly there is a balance to be struck and the few exam questions which perhaps are more easily searched online may not be suitable for OBE-style assessments. However, it is especially interesting to note that there has been recent confirmation from the faculty at Imperial that the distribution of marks for the online written exams did not differ substantially from previous years. These findings have since been published^10^ and although counter-intuitive, are mirrored in the literature where there is generally found to be no significant difference between exam performance when using OBEs versus closed-book formats.^11^ This supports the idea discussed above that clinical written exams are good candidates for an OBE format. It seems that instead of artificially inflating attainment, OBEs may allow students to prioritise their learning in a more meaningful and pragmatic way which can seemingly only be deemed a success. More research is clearly needed to bolster the case for more systemic adoption of OBEs in future, but this is certainly an intriguing and perhaps unexpected observation stemming from the coronavirus pandemic which may have implications for the future.

In the same way that clinical placements are difficult to facilitate remotely, so too are practical clinical examinations or OSCEs and most medical schools were forced to cancel or postpone these this year. At Imperial, students have now been informed that final year OSCEs next year will include 7 remote stations in an attempt to ensure their deliverability. This is likely to include online history-taking stations where examination findings such as breathing sounds and heart murmurs are to be played to students to help build a complete clinical picture and more closely mimic the bedside. This is yet another example of the COVID-19 pandemic accelerating the innovation of alternative educational and appraisal techniques.

Overall, the coronavirus crisis has unequivocally had a marked influence on medical education, particularly in terms of the delivery of teaching and assessment. However, as the world begins to emerge from this challenging period, it seems possible that this pandemic will leave lasting changes on these foundational elements of medical training. Such changes may include increased integration of ‘flipped learning’ and ‘telemedicine’ as means of content delivery as well as a re-imagination of current medical school examination systems. The common theme is that the COVID-19 pandemic has provided the catalyst required to diversify the format and delivery of both medical teaching and assessment and in doing so has depicted the intrinsic advantages of many of these contemporary approaches relative to their more conventional archetypes. Most of these educational changes were initially born out of urgency, but many will likely remain in more refined forms as preferred methods of teaching and assessment in the future.
